# Polymeric strontium ranelate nona­hydrate

**DOI:** 10.1107/S1600536811010099

**Published:** 2011-03-23

**Authors:** Kenny Stahl, Christian G. Frankaer, Anders C. Raffalt, Søren R. Sørensen, Jens E. T. Andersen

**Affiliations:** a Department of Chemistry, Technical University of Denmark, DK-2800 Lyngby, Denmark

## Abstract

The title compound, poly[[μ-aqua-tetra­aqua{μ-5-[bis­(carboxyl­atometh­yl)amino]-3-carboxyl­atomethyl-4-cyano­thio­phene-2-carboxyl­ato}distrontium(II)] tetra­hydrate], [Sr_2_(C_12_H_6_N_2_O_8_S)(H_2_O)_5_]·3.79H_2_O, crystallizes with nine- and eight-coordinated Sr^2+^ cations. They are bound to seven of the eight ranelate O atoms and five of the water mol­ecules. The SrO_8_ and SrO_9_ polyhedra are inter­connected by edge-sharing, forming hollow layers parallel to (011). The layers are, in turn, inter­connected by ranelate anions, forming a metal–organic framework (MOF) structure with channels along the *a* axis. The four water mol­ecules not coordinated to strontium are located in these channels and hydrogen bonded to each other and to the ranelates. Part of the water H atoms are disordered. The compound dehydrates very easily and 0.210 (4) water mol­ecules out of nine were lost during crystal mounting causing additional disorder in the water structure.

## Related literature

For the effect of strontium on osteroporosis, see Schrooten *et al.* (2003) ▶. For a patent describing the synthesis and powder diffraction pattern of the title compound, see Horvath *et al.* (2008 ▶). For related strontium carboxyl­ate structures, see, for example: Stahl *et al.* (2006 ▶). 
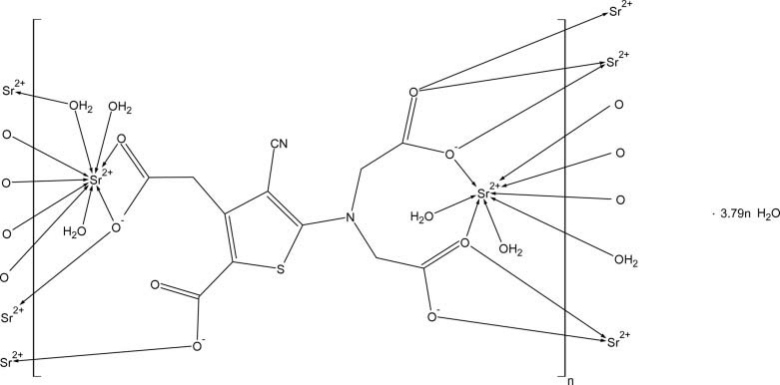


## Experimental

### 

#### Crystal data

[Sr_2_(C_12_H_6_N_2_O_8_S)(H_2_O)_5_]·3.79H_2_O
*M* *_r_* = 671.84
Triclinic, 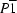

*a* = 8.3585 (3) Å
*b* = 12.3865 (5) Å
*c* = 12.6474 (5) Å
α = 109.880 (1)°
β = 97.148 (1)°
γ = 105.321 (1)°
*V* = 1154.00 (8) Å^3^
*Z* = 2
Mo *K*α radiation
μ = 4.80 mm^−1^
*T* = 120 K
0.15 × 0.10 × 0.07 mm

#### Data collection

Bruker SMART APEX diffractometer
Absorption correction: multi-scan (*SADABS*; Sheldrick, 2002 ▶) *T*_min_ = 0.574, *T*_max_ = 0.710
17404 measured reflections
6617 independent reflections
5375 reflections with *I* > 2σ(*I*)
*R*_int_ = 0.032

#### Refinement

*R*[*F* ^2^ > 2σ(*F* ^2^)] = 0.033
*wR*(*F* ^2^) = 0.075
*S* = 1.02
6617 reflections
375 parameters
21 restraints
H atoms treated by a mixture of independent and constrained refinement
Δρ_max_ = 1.48 e Å^−3^
Δρ_min_ = −1.23 e Å^−3^

### 

Data collection: *SMART* (Bruker, 1999 ▶); cell refinement: *SAINT-Plus* (Bruker, 1999 ▶); data reduction: *SAINT-Plus*; program(s) used to solve structure: *SHELXS97* (Sheldrick, 2008 ▶); program(s) used to refine structure: *SHELXL97* (Sheldrick, 2008 ▶); molecular graphics: *ORTEP-3* (Farrugia, 1997 ▶) and *ATOMS* (Dowty, 2000 ▶); software used to prepare material for publication: *SHELXL97*.

## Supplementary Material

Crystal structure: contains datablocks I, global. DOI: 10.1107/S1600536811010099/si2343sup1.cif

Structure factors: contains datablocks I. DOI: 10.1107/S1600536811010099/si2343Isup2.hkl

Additional supplementary materials:  crystallographic information; 3D view; checkCIF report

## Figures and Tables

**Table 1:** Selected bond lengths (Å)

Sr1—O8	2.4557 (18)
Sr1—O3^i^	2.4782 (19)
Sr1—O5	2.5234 (16)
Sr1—O7^ii^	2.6149 (19)
Sr1—O25	2.652 (2)
Sr1—O22	2.6560 (19)
Sr1—O27	2.657 (2)
Sr1—O8^ii^	2.7834 (17)
Sr2—O6^iii^	2.5452 (16)
Sr2—O23	2.5921 (18)
Sr2—O2^i^	2.6222 (17)
Sr2—O21	2.6445 (17)
Sr2—O6	2.6628 (16)
Sr2—O2^iv^	2.6848 (16)
Sr2—O1^iv^	2.6944 (17)
Sr2—O22	2.7108 (18)
Sr2—O5	2.7228 (16)

Symmetry codes: (i) 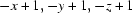; (ii) 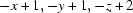; (iii) 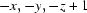; (iv) 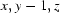.

**Table 2:** Hydrogen-bond geometry (Å, °)

*D*—H⋯*A*	*D*—H	H⋯*A*	*D*⋯*A*	*D*—H⋯*A*
O21—H21*A*⋯O25	0.81 (2)	1.98 (2)	2.781 (3)	169 (3)
O21—H21*B*⋯O24^iii^	0.85 (2)	1.93 (2)	2.765 (3)	169 (3)
O22—H22*A*⋯O2^i^	0.81 (2)	2.16 (3)	2.761 (2)	131 (3)
O22—H22*B*⋯O26^iv^	0.82 (2)	1.94 (2)	2.755 (3)	173 (3)
O23—H23*A*⋯O21^iii^	0.81 (2)	1.96 (2)	2.766 (3)	174 (4)
O23—H23*B*⋯O26^i^	0.80 (2)	2.11 (2)	2.867 (3)	159 (3)
O24—H24*A*⋯O1^v^	0.82 (2)	2.03 (2)	2.760 (3)	148 (3)
O24—H24*B*⋯O4	0.84 (2)	1.93 (2)	2.756 (3)	172 (3)
O25—H25*A*⋯N1^ii^	0.82 (2)	2.15 (2)	2.898 (3)	152 (3)
O25—H25*B*⋯O27^ii^	0.80 (2)	1.93 (2)	2.648 (3)	150 (4)
O26—H26*A*⋯O28^vi^	0.85 (2)	1.90 (2)	2.731 (3)	164 (5)
O26—H26*C*⋯N1	0.85 (2)	2.37 (4)	3.108 (3)	146 (5)
O27—H27*A*⋯O4^i^	0.83 (2)	1.79 (2)	2.615 (3)	172 (4)
O27—H27*B*⋯O29^vii^	0.82 (2)	1.94 (2)	2.727 (4)	160 (4)
O28—H28*A*⋯O24^v^	0.80 (2)	1.97 (2)	2.756 (3)	167 (4)
O28—H28*B*⋯O28^viii^	0.82 (2)	2.02 (2)	2.835 (4)	174 (8)
O28—H28*C*⋯O29	0.82 (2)	2.04 (3)	2.836 (4)	164 (7)
O29—H29*A*⋯O7	0.83 (2)	1.78 (2)	2.595 (3)	168 (7)
O29—H29*B*⋯O28	0.83 (2)	2.03 (3)	2.836 (4)	164 (7)

Symmetry codes: (i) 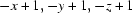; (ii) 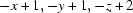; (iii) 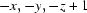; (iv) 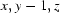; (v) 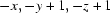; (vi) 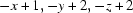; (vii) 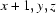; (viii) 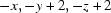.

**Fig. 1.**
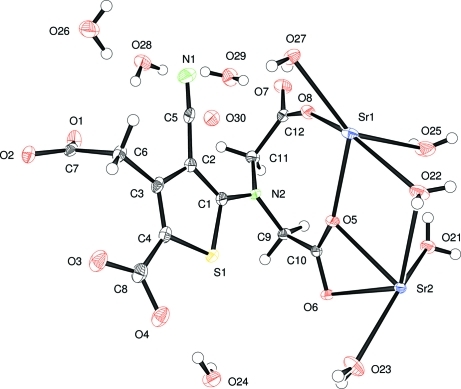
The asymmetric unit of (I) showing 50% probability displacement ellipsoids and the atomic numbering. Hydrogen atoms are represented by circles of arbitrary size and shows one consistent set of water H atoms.

**Fig. 2.**
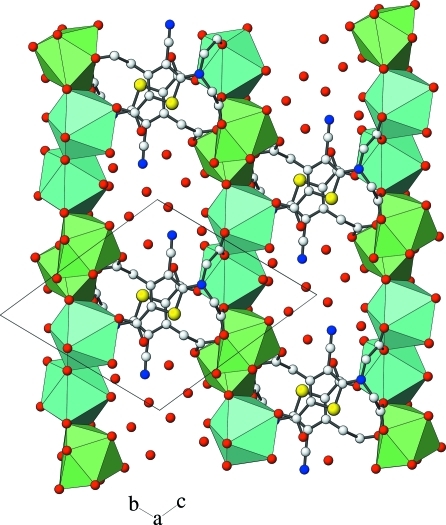
The crystal packing of (I) viewed down the a-axis. Hydrogen atoms are omitted for clarity.

**Fig. 3.**
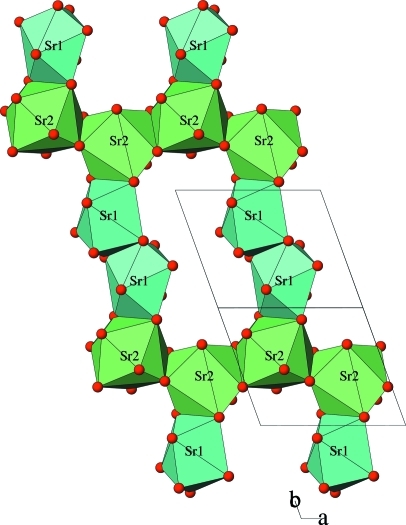
The polyhedral layer of (I) viewed down the (011) direction.

## supplementary crystallographic information

### Comment

In recent years it has been found that Sr has a significant influence on the development and growth of bone and the effect of dose on bone structure has been investigated in great detail (Schrooten *et al.* 2003). Strontium ranelate (5-[bis(carboxymethyl) amino]-3-carboxymetyl- 4-cyano-2-thiophenecarboxylate) is one promising pharmaceutical compound for treating osteoporosis marketed as Protelos^R^ by Servier (Horvath *et al.*, 2008). Strontium ranelate is known to form several hydrates with totally nine, eight, seven or four waters (Horvath *et al.*, 2008). The initial dehydration observed here results from an expulsion of O27 or O29 and migration of the remaining water to site O30. As a consequence Sr1 is partially seven-coordinated (c.f. Table 1). The water hydrogen sites connected to O26, O28 and O29 are disordered. In essence, the alternating hydrogen bonding scheme between O28 and O29 is transmitted to a partial O26 - O28 hydrogen bond, and leaves H26B and H29C without hydrogen bond acceptors (c.f. Table 2).

### Experimental

Strontium ranelate nona hydrate of 97% purity (Clauson-Kaas A/S) was recrystallized at different temperatures. Recrystallization at temperatures above 353 K appeared to produce the crystals of better quality. Upon cooling to room temperature large crystals of millimeter dimensions were obtained in the saturated solution. However, when the crystals were removed from the solution they rapidly degraded into smaller units of micron dimension. The smaller crystals showed out to contain less crystal water, as compared to the large crystals, presumably seven or five water molecules per formula unit. Thus, wet crystals were quickly transferred to the goniometer for X-ray data collection at 120 K. Several crystal were tried before an acceptable structure refinement was achieved. For all cases of lower quality data the SOF of O30 was about 0.3, confirming its role in the initial dehydration of strontium ranelate and the deterioration of the crystals.

### Refinement

The H atoms of the CH_2_ groups were placed in calculated positions with C—H = 0.99, and refined as riding atoms. The H atoms of the water molecules were located in difference Fourier maps and refined with restrained O—H distances of 0.82 (2) Å. The H atoms of the partially occupied O30 (SOF=0.210 (4)) could not be located. The H displacement parameters were set to 1.2 (CH_2_) or 1.5 (H_2_O) times *U*_eq_ of the corresponding C or O atoms.

### Crystal data

**Table:**

[Sr_2_(C_12_H_6_N_2_O_8_S)(H_2_O)_5_]·3.79H_2_O	*Z* = 2
*M**_r_* = 671.84	*F*(000) = 671.8
Triclinic, *P*1	*D*_x_ = 1.933 Mg m^−^^3^
Hall symbol: -P 1	Mo *K*α radiation, λ = 0.71073 Å
*a* = 8.3585 (3) Å	Cell parameters from 6752 reflections
*b* = 12.3865 (5) Å	θ = 2.6–30.6°
*c* = 12.6474 (5) Å	µ = 4.80 mm^−^^1^
α = 109.880 (1)°	*T* = 120 K
β = 97.148 (1)°	Tabular, colorless
γ = 105.321 (1)°	0.15 × 0.10 × 0.07 mm
*V* = 1154.00 (8) Å^3^	

### Data collection

**Table:**

Bruker SMART APEX diffractometer	6617 independent reflections
Radiation source: fine-focus sealed tube	5375 reflections with *I* > 2σ(*I*)
graphite	*R*_int_ = 0.032
ω scan, frame data integration	θ_max_ = 30.9°, θ_min_ = 2.6°
Absorption correction: multi-scan (*SADABS*; Sheldrick, 2002)	*h* = −12→12
*T*_min_ = 0.574, *T*_max_ = 0.710	*k* = −17→17
17404 measured reflections	*l* = −18→18

### Refinement

**Table:**

Refinement on *F*^2^	Primary atom site location: structure-invariant direct methods
Least-squares matrix: full	Secondary atom site location: difference Fourier map
*R*[*F*^2^ > 2σ(*F*^2^)] = 0.033	Hydrogen site location: inferred from neighbouring sites
*wR*(*F*^2^) = 0.075	H atoms treated by a mixture of independent and constrained refinement
*S* = 1.02	*w* = 1/[σ^2^(*F*_o_^2^) + (0.0383*P*)^2^] where *P* = (*F*_o_^2^ + 2*F*_c_^2^)/3
6617 reflections	(Δ/σ)_max_ = 0.002
375 parameters	Δρ_max_ = 1.48 e Å^−^^3^
21 restraints	Δρ_min_ = −1.23 e Å^−^^3^

### Special details

**Table:**

Geometry. All e.s.d.'s (except the e.s.d. in the dihedral angle between two l.s. planes) are estimated using the full covariance matrix. The cell e.s.d.'s are taken into account individually in the estimation of e.s.d.'s in distances, angles and torsion angles; correlations between e.s.d.'s in cell parameters are only used when they are defined by crystal symmetry. An approximate (isotropic) treatment of cell e.s.d.'s is used for estimating e.s.d.'s involving l.s. planes.
Refinement. Refinement of *F*^2^ against ALL reflections. The weighted *R*-factor *wR* and goodness of fit *S* are based on *F*^2^, conventional *R*-factors *R* are based on *F*, with *F* set to zero for negative *F*^2^. The threshold expression of *F*^2^ > 2σ(*F*^2^) is used only for calculating *R*-factors(gt) *etc*. and is not relevant to the choice of reflections for refinement. *R*-factors based on *F*^2^ are statistically about twice as large as those based on *F*, and *R*- factors based on ALL data will be even larger.

### Fractional atomic coordinates and isotropic or equivalent isotropic displacement parameters (Å^2^)

**Table:**

	*x*	*y*	*z*	*U*_iso_*/*U*_eq_	Occ. (<1)
Sr1	0.52281 (3)	0.38668 (2)	0.836097 (18)	0.01665 (6)	
Sr2	0.26745 (3)	0.029136 (18)	0.557006 (17)	0.01072 (6)	
S1	0.13839 (8)	0.40229 (5)	0.50122 (5)	0.01584 (12)	
C1	0.1878 (3)	0.4919 (2)	0.6477 (2)	0.0160 (5)	
C2	0.3059 (3)	0.6056 (2)	0.6709 (2)	0.0183 (5)	
C3	0.3561 (3)	0.6182 (2)	0.5698 (2)	0.0168 (5)	
C4	0.2738 (3)	0.5158 (2)	0.4721 (2)	0.0169 (5)	
C5	0.3781 (4)	0.7012 (2)	0.7831 (2)	0.0237 (6)	
N1	0.4382 (4)	0.7807 (2)	0.8712 (2)	0.0373 (7)	
C6	0.4862 (3)	0.7305 (2)	0.5736 (2)	0.0187 (5)	
H6A	0.5431	0.7077	0.5091	0.022*	
H6B	0.5745	0.7663	0.6470	0.022*	
C7	0.4063 (3)	0.8258 (2)	0.5642 (2)	0.0140 (4)	
O1	0.3026 (2)	0.85078 (16)	0.62454 (15)	0.0218 (4)	
O2	0.4503 (2)	0.87870 (15)	0.49792 (15)	0.0168 (3)	
C8	0.2835 (3)	0.4871 (2)	0.3498 (2)	0.0208 (5)	
O3	0.3592 (3)	0.57144 (17)	0.32159 (17)	0.0293 (4)	
O4	0.2095 (3)	0.37659 (16)	0.28052 (16)	0.0253 (4)	
N2	0.1127 (3)	0.44571 (17)	0.71912 (16)	0.0138 (4)	
C9	0.0097 (3)	0.3176 (2)	0.6705 (2)	0.0147 (4)	
H9A	−0.0850	0.3036	0.6067	0.018*	
H9B	−0.0415	0.2978	0.7309	0.018*	
C10	0.1092 (3)	0.2303 (2)	0.62385 (19)	0.0125 (4)	
O5	0.2644 (2)	0.25859 (14)	0.67064 (14)	0.0150 (3)	
O6	0.0259 (2)	0.13041 (14)	0.54187 (13)	0.0141 (3)	
C11	0.0937 (3)	0.5241 (2)	0.82968 (19)	0.0167 (5)	
H11A	−0.0207	0.4870	0.8398	0.020*	
H11B	0.0977	0.6033	0.8259	0.020*	
C12	0.2261 (3)	0.5475 (2)	0.9357 (2)	0.0177 (5)	
O7	0.2069 (3)	0.60995 (17)	1.03249 (15)	0.0266 (4)	
O8	0.3467 (2)	0.50536 (16)	0.92535 (15)	0.0213 (4)	
O21	0.1336 (2)	0.04188 (16)	0.73788 (14)	0.0177 (4)	
H21A	0.195 (3)	0.096 (2)	0.7968 (19)	0.027*	
H21B	0.111 (4)	−0.021 (2)	0.753 (3)	0.027*	
O22	0.5486 (2)	0.16837 (18)	0.73157 (15)	0.0218 (4)	
H22A	0.608 (4)	0.162 (3)	0.686 (2)	0.033*	
H22B	0.572 (4)	0.129 (3)	0.768 (3)	0.033*	
O23	0.2011 (2)	−0.02488 (19)	0.33592 (16)	0.0246 (4)	
H23A	0.102 (3)	−0.035 (3)	0.311 (3)	0.037*	
H23B	0.249 (4)	−0.006 (3)	0.291 (2)	0.037*	
O24	−0.0724 (3)	0.17943 (17)	0.24069 (16)	0.0233 (4)	
H24A	−0.138 (4)	0.198 (3)	0.282 (3)	0.035*	
H24B	0.015 (3)	0.240 (2)	0.260 (3)	0.035*	
O25	0.3787 (3)	0.23012 (19)	0.92443 (16)	0.0299 (5)	
H25A	0.430 (4)	0.204 (3)	0.965 (3)	0.045*	
H25B	0.316 (4)	0.259 (3)	0.958 (3)	0.045*	
O26	0.6112 (3)	1.0182 (2)	0.83803 (19)	0.0321 (5)	
H26A	0.686 (5)	1.030 (5)	0.897 (3)	0.048*	0.67
H26B	0.549 (6)	1.037 (5)	0.885 (4)	0.048*	0.67
H26C	0.532 (5)	0.952 (3)	0.823 (5)	0.048*	0.67
O27	0.7186 (3)	0.6180 (2)	0.91336 (19)	0.0194 (6)	0.790 (4)
H27A	0.740 (5)	0.626 (4)	0.854 (2)	0.029*	0.79
H27B	0.811 (4)	0.640 (4)	0.959 (3)	0.029*	0.79
O28	0.1058 (3)	0.92663 (19)	0.99457 (17)	0.0302 (5)	
H28A	0.111 (5)	0.896 (3)	0.9290 (18)	0.045*	
H28B	0.041 (8)	0.966 (6)	1.000 (7)	0.045*	0.50
H28C	0.059 (8)	0.873 (5)	1.015 (6)	0.045*	0.50
O29	0.0120 (4)	0.7429 (3)	1.0829 (2)	0.0287 (7)	0.790 (4)
H29A	0.073 (7)	0.701 (5)	1.058 (6)	0.043*	0.53
H29B	0.045 (9)	0.806 (4)	1.071 (6)	0.043*	0.53
H29C	−0.024 (10)	0.732 (8)	1.016 (3)	0.043*	0.53
O30	−0.0951 (12)	0.6963 (8)	0.9739 (8)	0.023 (2)*	0.210 (4)

### Atomic displacement parameters (Å^2^)

**Table:**

	*U*^11^	*U*^22^	*U*^33^	*U*^12^	*U*^13^	*U*^23^
Sr1	0.01489 (12)	0.02075 (12)	0.00915 (10)	0.00204 (9)	0.00133 (8)	0.00307 (9)
Sr2	0.01086 (11)	0.01068 (10)	0.00940 (10)	0.00340 (8)	0.00144 (7)	0.00290 (8)
S1	0.0195 (3)	0.0115 (3)	0.0149 (3)	0.0032 (2)	0.0029 (2)	0.0051 (2)
C1	0.0158 (12)	0.0144 (11)	0.0177 (11)	0.0064 (9)	−0.0008 (9)	0.0067 (9)
C2	0.0197 (12)	0.0133 (11)	0.0215 (12)	0.0061 (10)	−0.0004 (10)	0.0078 (10)
C3	0.0133 (11)	0.0122 (11)	0.0255 (12)	0.0049 (9)	0.0006 (9)	0.0087 (10)
C4	0.0164 (12)	0.0138 (11)	0.0241 (12)	0.0060 (9)	0.0057 (10)	0.0105 (10)
C5	0.0287 (15)	0.0139 (12)	0.0256 (13)	0.0010 (11)	−0.0027 (11)	0.0120 (11)
N1	0.0485 (17)	0.0204 (12)	0.0261 (13)	−0.0078 (11)	−0.0058 (12)	0.0083 (10)
C6	0.0129 (11)	0.0152 (12)	0.0301 (13)	0.0046 (9)	0.0032 (10)	0.0122 (10)
C7	0.0120 (11)	0.0104 (10)	0.0167 (11)	0.0015 (8)	0.0008 (9)	0.0042 (9)
O1	0.0269 (10)	0.0231 (9)	0.0262 (9)	0.0140 (8)	0.0147 (8)	0.0152 (8)
O2	0.0174 (9)	0.0160 (8)	0.0201 (8)	0.0055 (7)	0.0065 (7)	0.0101 (7)
C8	0.0220 (13)	0.0200 (13)	0.0260 (13)	0.0100 (11)	0.0119 (11)	0.0111 (11)
O3	0.0386 (12)	0.0209 (10)	0.0317 (11)	0.0058 (9)	0.0167 (9)	0.0141 (9)
O4	0.0348 (11)	0.0179 (9)	0.0252 (10)	0.0072 (8)	0.0152 (8)	0.0092 (8)
N2	0.0185 (10)	0.0084 (9)	0.0109 (9)	0.0030 (8)	0.0002 (7)	0.0016 (7)
C9	0.0142 (11)	0.0121 (11)	0.0150 (11)	0.0029 (9)	0.0014 (9)	0.0038 (9)
C10	0.0157 (11)	0.0109 (10)	0.0106 (10)	0.0022 (9)	0.0027 (8)	0.0056 (8)
O5	0.0125 (8)	0.0144 (8)	0.0140 (8)	0.0033 (6)	−0.0023 (6)	0.0032 (6)
O6	0.0140 (8)	0.0104 (8)	0.0127 (8)	0.0016 (6)	−0.0014 (6)	0.0016 (6)
C11	0.0195 (12)	0.0164 (11)	0.0125 (11)	0.0096 (10)	0.0008 (9)	0.0017 (9)
C12	0.0235 (13)	0.0113 (11)	0.0151 (11)	0.0023 (9)	0.0003 (9)	0.0055 (9)
O7	0.0378 (12)	0.0255 (10)	0.0124 (8)	0.0109 (9)	0.0040 (8)	0.0028 (7)
O8	0.0213 (9)	0.0194 (9)	0.0199 (9)	0.0077 (7)	−0.0036 (7)	0.0056 (7)
O21	0.0200 (9)	0.0184 (9)	0.0120 (8)	0.0043 (7)	0.0025 (7)	0.0046 (7)
O22	0.0186 (9)	0.0305 (10)	0.0139 (9)	0.0109 (8)	0.0024 (7)	0.0043 (8)
O23	0.0172 (9)	0.0421 (12)	0.0195 (9)	0.0106 (9)	0.0070 (8)	0.0164 (9)
O24	0.0270 (11)	0.0201 (9)	0.0198 (9)	0.0028 (8)	0.0098 (8)	0.0066 (8)
O25	0.0413 (13)	0.0248 (11)	0.0151 (9)	0.0009 (9)	0.0046 (9)	0.0060 (8)
O26	0.0299 (12)	0.0322 (12)	0.0283 (11)	0.0011 (10)	0.0029 (9)	0.0131 (10)
O27	0.0243 (13)	0.0161 (11)	0.0129 (11)	0.0003 (9)	0.0054 (9)	0.0042 (9)
O28	0.0422 (14)	0.0295 (12)	0.0163 (9)	0.0133 (10)	0.0022 (9)	0.0060 (9)
O29	0.0313 (15)	0.0330 (15)	0.0249 (14)	0.0161 (12)	0.0070 (11)	0.0107 (12)

### Geometric parameters (Å, °)

**Table:**

Sr1—O8	2.4557 (18)	N2—C11	1.462 (3)
Sr1—O3^i^	2.4782 (19)	C9—C10	1.540 (3)
Sr1—O5	2.5234 (16)	C9—H9A	0.9900
Sr1—O7^ii^	2.6149 (19)	C9—H9B	0.9900
Sr1—O25	2.652 (2)	C10—O5	1.258 (3)
Sr1—O22	2.6560 (19)	C10—O6	1.258 (3)
Sr1—O27	2.657 (2)	C11—C12	1.517 (3)
Sr1—O8^ii^	2.7834 (17)	C11—H11A	0.9900
Sr2—O6^iii^	2.5452 (16)	C11—H11B	0.9900
Sr2—O23	2.5921 (18)	C12—O8	1.253 (3)
Sr2—O2^i^	2.6222 (17)	C12—O7	1.262 (3)
Sr2—O21	2.6445 (17)	O21—H21A	0.809 (18)
Sr2—O6	2.6628 (16)	O21—H21B	0.845 (17)
Sr2—O2^iv^	2.6848 (16)	O22—H22A	0.808 (18)
Sr2—O1^iv^	2.6944 (17)	O22—H22B	0.819 (17)
Sr2—O22	2.7108 (18)	O23—H23A	0.807 (18)
Sr2—O5	2.7228 (16)	O23—H23B	0.798 (18)
S1—C1	1.733 (2)	O24—H24A	0.823 (18)
S1—C4	1.735 (2)	O24—H24B	0.837 (18)
C1—N2	1.358 (3)	O25—H25A	0.819 (18)
C1—C2	1.398 (3)	O25—H25B	0.796 (18)
C2—C5	1.431 (4)	O26—H26A	0.850 (19)
C2—C3	1.439 (4)	O26—H26B	0.849 (19)
C3—C4	1.368 (3)	O26—H26C	0.85 (2)
C3—C6	1.504 (3)	O27—H27A	0.827 (19)
C4—C8	1.482 (4)	O27—H27B	0.823 (19)
C5—N1	1.148 (3)	O28—H28A	0.799 (18)
C6—C7	1.532 (3)	O28—H28B	0.82 (2)
C6—H6A	0.9900	O28—H28C	0.82 (2)
C6—H6B	0.9900	O29—H29A	0.83 (2)
C7—O1	1.256 (3)	O29—H29B	0.83 (2)
C7—O2	1.261 (3)	O29—H29C	0.82 (2)
C8—O3	1.257 (3)	O27—O30^v^	1.534 (10)
C8—O4	1.275 (3)	O29—O30	1.383 (10)
N2—C9	1.456 (3)		
			
O8—Sr1—O3^i^	117.69 (6)	O22—Sr2—O5	67.10 (5)
O8—Sr1—O5	87.39 (5)	C1—S1—C4	92.44 (12)
O3^i^—Sr1—O5	81.32 (6)	N2—C1—C2	130.8 (2)
O8—Sr1—O7^ii^	118.97 (6)	N2—C1—S1	119.08 (17)
O3^i^—Sr1—O7^ii^	101.51 (7)	C2—C1—S1	110.09 (19)
O5—Sr1—O7^ii^	146.34 (5)	C1—C2—C5	125.2 (2)
O8—Sr1—O25	85.98 (7)	C1—C2—C3	113.4 (2)
O3^i^—Sr1—O25	150.13 (6)	C5—C2—C3	121.3 (2)
O5—Sr1—O25	81.94 (6)	C4—C3—C2	111.8 (2)
O7^ii^—Sr1—O25	79.69 (7)	C4—C3—C6	124.9 (2)
O8—Sr1—O22	147.38 (6)	C2—C3—C6	123.3 (2)
O3^i^—Sr1—O22	83.58 (6)	C3—C4—C8	131.1 (2)
O5—Sr1—O22	70.82 (5)	C3—C4—S1	112.22 (19)
O7^ii^—Sr1—O22	76.14 (6)	C8—C4—S1	116.67 (18)
O25—Sr1—O22	67.59 (6)	N1—C5—C2	177.5 (3)
O8—Sr1—O27	74.49 (7)	C3—C6—C7	112.26 (19)
O3^i^—Sr1—O27	70.14 (7)	C3—C6—H6A	109.2
O5—Sr1—O27	132.72 (6)	C7—C6—H6A	109.2
O7^ii^—Sr1—O27	77.82 (7)	C3—C6—H6B	109.2
O25—Sr1—O27	137.67 (6)	C7—C6—H6B	109.2
O22—Sr1—O27	138.07 (7)	H6A—C6—H6B	107.9
O8—Sr1—O8^ii^	70.87 (6)	O1—C7—O2	122.2 (2)
O3^i^—Sr1—O8^ii^	130.40 (6)	O1—C7—C6	119.1 (2)
O5—Sr1—O8^ii^	147.09 (6)	O2—C7—C6	118.7 (2)
O7^ii^—Sr1—O8^ii^	48.19 (5)	O3—C8—O4	125.2 (2)
O25—Sr1—O8^ii^	72.41 (6)	O3—C8—C4	118.9 (2)
O22—Sr1—O8^ii^	115.58 (5)	O4—C8—C4	115.9 (2)
O27—Sr1—O8^ii^	65.82 (6)	C1—N2—C9	117.63 (19)
O6^iii^—Sr2—O23	69.11 (6)	C1—N2—C11	121.82 (19)
O6^iii^—Sr2—O2^i^	138.88 (5)	C9—N2—C11	118.5 (2)
O23—Sr2—O2^i^	71.52 (6)	N2—C9—C10	114.24 (19)
O6^iii^—Sr2—O21	79.58 (5)	N2—C9—H9A	108.7
O23—Sr2—O21	145.03 (6)	C10—C9—H9A	108.7
O2^i^—Sr2—O21	141.51 (5)	N2—C9—H9B	108.7
O6^iii^—Sr2—O6	68.34 (6)	C10—C9—H9B	108.7
O23—Sr2—O6	81.03 (5)	H9A—C9—H9B	107.6
O2^i^—Sr2—O6	116.53 (5)	O5—C10—O6	123.3 (2)
O21—Sr2—O6	73.03 (5)	O5—C10—C9	119.92 (19)
O6^iii^—Sr2—O2^iv^	96.94 (5)	O6—C10—C9	116.7 (2)
O23—Sr2—O2^iv^	81.54 (6)	N2—C11—C12	115.7 (2)
O2^i^—Sr2—O2^iv^	65.68 (6)	N2—C11—H11A	108.3
O21—Sr2—O2^iv^	118.40 (5)	C12—C11—H11A	108.3
O6—Sr2—O2^iv^	160.37 (5)	N2—C11—H11B	108.3
O6^iii^—Sr2—O1^iv^	79.58 (5)	C12—C11—H11B	108.3
O23—Sr2—O1^iv^	116.26 (6)	H11A—C11—H11B	107.4
O2^i^—Sr2—O1^iv^	108.25 (5)	O8—C12—O7	122.9 (2)
O21—Sr2—O1^iv^	71.00 (5)	O8—C12—C11	120.5 (2)
O6—Sr2—O1^iv^	135.19 (5)	O7—C12—C11	116.7 (2)
O2^iv^—Sr2—O1^iv^	48.34 (5)	H21A—O21—H21B	106 (3)
O6^iii^—Sr2—O22	156.57 (6)	H22A—O22—H22B	104 (3)
O23—Sr2—O22	133.65 (6)	H23A—O23—H23B	104 (3)
O2^i^—Sr2—O22	62.33 (5)	H24A—O24—H24B	108 (3)
O21—Sr2—O22	79.68 (6)	H25A—O25—H25B	109 (4)
O6—Sr2—O22	115.27 (5)	H26A—O26—H26B	87 (5)
O2^iv^—Sr2—O22	83.49 (5)	H26A—O26—H26C	108 (5)
O1^iv^—Sr2—O22	83.47 (6)	H26B—O26—H26C	75 (5)
O6^iii^—Sr2—O5	114.87 (5)	H27A—O27—H27B	107 (4)
O23—Sr2—O5	109.66 (6)	H28A—O28—H28B	111 (6)
O2^i^—Sr2—O5	89.11 (5)	H28A—O28—H28C	109 (6)
O21—Sr2—O5	69.47 (5)	H28B—O28—H28C	103 (7)
O6—Sr2—O5	48.56 (5)	H29A—O29—H29B	107 (7)
O2^iv^—Sr2—O5	148.19 (5)	H29A—O29—H29C	87 (6)
O1^iv^—Sr2—O5	133.93 (5)	H29B—O29—H29C	68 (6)

Symmetry codes: (i) −*x*+1, −*y*+1, −*z*+1; (ii) −*x*+1, −*y*+1, −*z*+2; (iii) −*x*, −*y*, −*z*+1; (iv) *x*, *y*−1, *z*; (v) *x*+1, *y*, *z*.

### Hydrogen-bond geometry (Å, °)

**Table:**

*D*—H···*A*	*D*—H	H···*A*	*D*···*A*	*D*—H···*A*
O21—H21A···O25	0.81 (2)	1.98 (2)	2.781 (3)	169 (3)
O21—H21B···O24^iii^	0.85 (2)	1.93 (2)	2.765 (3)	169 (3)
O22—H22A···O2^i^	0.81 (2)	2.16 (3)	2.761 (2)	131 (3)
O22—H22B···O26^iv^	0.82 (2)	1.94 (2)	2.755 (3)	173 (3)
O23—H23A···O21^iii^	0.81 (2)	1.96 (2)	2.766 (3)	174 (4)
O23—H23B···O26^i^	0.80 (2)	2.11 (2)	2.867 (3)	159 (3)
O24—H24A···O1^vi^	0.82 (2)	2.03 (2)	2.760 (3)	148 (3)
O24—H24B···O4	0.84 (2)	1.93 (2)	2.756 (3)	172 (3)
O25—H25A···N1^ii^	0.82 (2)	2.15 (2)	2.898 (3)	152 (3)
O25—H25B···O27^ii^	0.80 (2)	1.93 (2)	2.648 (3)	150 (4)
O26—H26A···O28^vii^	0.85 (2)	1.90 (2)	2.731 (3)	164 (5)
O26—H26C···N1	0.85 (2)	2.37 (4)	3.108 (3)	146 (5)
O27—H27A···O4^i^	0.83 (2)	1.79 (2)	2.615 (3)	172 (4)
O27—H27B···O29^v^	0.82 (2)	1.94 (2)	2.727 (4)	160 (4)
O28—H28A···O24^vi^	0.80 (2)	1.97 (2)	2.756 (3)	167 (4)
O28—H28B···O28^viii^	0.82 (2)	2.02 (2)	2.835 (4)	174 (8)
O28—H28C···O29	0.82 (2)	2.04 (3)	2.836 (4)	164 (7)
O29—H29A···O7	0.83 (2)	1.78 (2)	2.595 (3)	168 (7)
O29—H29B···O28	0.83 (2)	2.03 (3)	2.836 (4)	164 (7)

Symmetry codes:  (iii) −*x*, −*y*, −*z*+1; (i) −*x*+1, −*y*+1, −*z*+1; (iv) *x*, *y*−1, *z*; (vi) −*x*, −*y*+1, −*z*+1; (ii) −*x*+1, −*y*+1, −*z*+2; (vii) −*x*+1, −*y*+2, −*z*+2; (v) *x*+1, *y*, *z*; (viii) −*x*, −*y*+2, −*z*+2.
